# Association between Statin Use and Risk of Parkinson’s Disease: Evidence from 18 Observational Studies Comprising 3.7 Million Individuals

**DOI:** 10.3390/jpm12050825

**Published:** 2022-05-19

**Authors:** Chieh-Chen Wu, Md. Mohaimenul Islam, An-Jen Lee, Chun-Hsien Su, Yung-Ching Weng, Chih-Yang Yeh, Hsun-Hua Lee, Ming-Chin Lin

**Affiliations:** 1 Department of Healthcare Information and Management, School of Health Technology, Ming Chuan University, Taipei 333, Taiwan; wjc4@ulive.pccu.edu.tw (C.-C.W.); anjenlee@mail.mcu.edu.tw (A.-J.L.); kimweng@mail.mcu.edu.tw (Y.-C.W.); 2 Department of Exercise and Health Promotion, College of Kinesiology and Health, Chinese Culture University, Taipei 111369, Taiwan; chsu@ulive.pccu.edu.tw; 3 AESOP Technology, Taipei 105, Taiwan; d610106004@tmu.edu.tw; 4 Southeast Asian, Cross-Strait and Overseas Student Institute, Ming Chuan University, Taipei 333, Taiwan; 5 Graduate Institute of Sport Coaching Science, College of Kinesiology and Health, Chinese Culture University, Taipei 11114, Taiwan; 6 Graduate Institute of Biomedical Informatics, College of Medical Science and Technology, Taipei Medical University, Taipei 106339, Taiwan; d610104001@tmu.edu.tw; 7 Department of Neurology, Taipei Medical University Hospital, Taipei Medical University, Taipei 11031, Taiwan; 8 Department of Neurology, School of Medicine, College of Medicine, Taipei Medical University, Taipei 11031, Taiwan; 9 Dizziness and Balance Disorder Center, Shuang Ho Hospital, Taipei Medical University, New Taipei City 23561, Taiwan; 10 Department of Neurosurgery, Shuang Ho Hospital, Taipei Medical University, New Taipei City 235041, Taiwan; 11 Taipei Neuroscience Institute, Taipei Medical University, Taipei 110301, Taiwan

**Keywords:** statins, Parkinson’s disease, meta-analysis, observational studies

## Abstract

The potential impact of statins on the risk of Parkinson’s disease (PD) is still controversial; therefore, we conducted a comprehensive meta-analysis of observational studies to examine the effect of statin use on the risk of PD. We searched electronic databases, such as PubMed, EMBASE, Scopus, and Web of Science, for articles published between 1 January 2000 and 15 March 2022. Cohort studies which examined the association between statins and PD risk in the general population were also included. Two authors assessed the data and extracted all potential information for analysis. Random effects meta-analyses were performed to measure the risk ratio (RR) and 95% confidence intervals (CIs). Eighteen cohort studies including 3.7 million individuals with 31,153 PD participants were identified. In statin users, compared with non-users, the RR for PD was 0.79 (95% CI: 0.68–0.91). In a subgroup analysis of PD, this association was observed with medium and high quality, and the studies were adjusted for age, gender, and smoking status. When the data were stratified according to the duration of exposure, long-duration statin use was associated with a decreased risk of PD (RR = 0.49; 95% CI: 0.26–0.92). There was no significant decrease in the risk of PD in short-term statin users (RR = 0.94; 95% CI: 0.67–1.31). Moreover, no significant difference in the reduction in the risk of PD was observed between men (RR = 0.80; 95% CI: 0.75–0.86) and women (RR = 0.80; 95% CI: 0.75–0.86). Although our findings confirm a reduction in the PD risk associated with statin treatment and suggest that statins play a clinically favorable role, these findings should be interpreted with caution. Future randomized control trials with an ad hoc design are needed to confirm the potential utility of statins in reducing the risk of PD.

## 1. Introduction

Parkinson’s disease (PD) is one of the major causes of death and disability globally [1]. More than 6.1 million individuals currently live with PD, compared with 2.1 million in 1990 [2]. Previous global burden studies have reported that prevalence and death rates vary across different countries, similar to what is reported for dementia [3]. There is growing evidence concerning different risk and protective factors, but age is the most important risk factor for PD. However, previous research highlights that a variety of occupational and environmental factors, such as pesticide exposure [4,5,6,7,8,9], metals [6,8,10,11], exposure to farm animals, and living on a farm [12], also appear to be associated with PD.

Statins, which are also called HMG-CoA reductase inhibitors, are considered a mainstay therapy in the management of dyslipidemia. They also have proven cardiovascular benefits, including illness and mortality reduction [13,14,15,16]. Statins are mainly recommended for patients who need to bring their cholesterol down to the normal range. Statins are considered a safe medication even for long-term use. Growing evidence has shown the beneficial effect of statins on cancer risk reduction [17,18], cancer survival [19], and dementia risk reduction [20]. However, there has been recent literature on the use of statin to decrease the risk of PD. These studies include geographically diverse prospective cohorts and different types of statins (lipophilic and hydrophilic). The precise mechanism of this significant reduction is still unknown, but several biological mechanisms have been proposed to explain a possible relationship between statin use and the risk of PD. These include a reduction in the production of tumor necrosis factor-alfa (TNF), as well as a reduction in dopamine 3,4-dihydroxyphenylacetic acid and homovanillic acid [21].

The potential effect of statin therapy on PD risk reduction is not conclusive. To our knowledge, six meta-analyses have evaluated the association between statins and the risk of PD [22,23,24,25,26,27], but these studies had limited power to draw solid conclusions. However, it is important to mention that previous meta-analyses have used a relatively modest sample size and have not provided substantial subgroups and sensitivity analyses. We therefore carried out an updated systematic review and meta-analysis of observational studies examining the association between statin use and the risk of PD. We believe that the clarification of the magnitude of the risk of PD associated with statins might have important clinical implications for future strategies for the prevention and treatment of PD.

## 2. Methods

We conducted this systematic review and network meta-analysis according to the Preferred Reporting Items for Systematic Reviews and Meta-Analyses (PRISMA) guidelines [28].

### 2.1. Data Sources and Search Strategies

Popular electronic databases, such as PubMed, Embase, Scopus, and Web of Science, were searched for articles published between 1 January 2000 and 15 March 2022. Database searches were conducted by two experts’ independent authors with predefined strategies, and disagreements were resolved by the principal investigator. The following keywords were used to search articles: “statin(s)” OR “HMG-CoA reductase inhibitor(s)” OR “lipid-lowering drug(s)” OR “lipid-lowering agent(s)” OR “simvastatin” OR “atorvastatin” OR “pravastatin” OR “Fluvastatin” OR “rosuvastatin” OR lovastatin” AND (“Parkinson disease” OR “Parkinson’s disease” OR “PD”). Moreover, the reference lists of the included studies and previous reviews were screened for more studies that could potentially be included.

### 2.2. Eligibility Criteria

The criteria for the inclusion of a potential study are as follows: (1) the study was an observational study (case–control and cohort study); (2) all or a subset of the participants had a history of PD; (3) the study evaluated statin therapies through a comparison between statin users and non-users; (4) the treatment duration was at least three months. Studies were excluded if they were published as reviews or case reports, or if they were not in English.

### 2.3. Data Extraction

A standard protocol was used to extract data from the included studies. The same two authors extracted data regarding study characteristics (author, publication year, country, study design, length of follow-up, total number of participants, number of PD patients, outcomes), patient characteristics (gender, mean/median age in years), inclusion criteria for statin users, inclusion criteria for PD patients, and adjusted variables such as the comorbidity and concomitant drug use.

### 2.4. Study Quality Assessment

Since all of the included studies were observational studies, the risk of bias in the included studies was assessed using the Newcastle–Ottawa Scale (NOS) [29]. The NOS contains eight items, categorized into three domains up to a maximum of nine points for the least risk of bias: (1) selection of study (four points); (2) comparability (two points); and (3) ascertainment of exposure (case–control study)/outcomes (cohort study) (three points) for the observational epidemiological studies, respectively. We considered a study as high, medium, and low if the total scores were 9, 7–8, and less than 7.

### 2.5. Statistical Analysis

The primary outcome of interest was the PD risk. We calculated a random-effects estimate based on the DerSimoneon and Laird method [30]. This is a simple and widely used method, due to the virtue that it is always qualitatively consistent with the heterogeneity test based on the Q statistic. The standard errors of the study-specific estimates were adjusted to incorporate a measure of the extent of variation (τ^2^), or heterogeneity (I^2^), among the intervention effects observed in different studies [31]. The relative risk (RR) with a 95% CI was used to measure the association of use of statins vs. no use of statins with PD. All *p* values were obtained from 2-sided tests, and effect sizes were considered statistically significant at *p* < 0.05. All effect sizes included in the pooled analyses were taken from a fully adjusted multivariable model.

Subgroup analyses were also conducted to assess possible sources of heterogeneity and to determine whether several confounding and clinical factors were associated with any significant differences in the outcome. Those factors were the (a) study design; (b) regional impact; (c) quality of a study; (d) study year; € whether the study was adjusted for age, gender, and smoking status; and (f) the various type(s) of statins used in the study. We assessed the heterogeneity among the studies using a *p*-value calculated using χ^2^ statistics and I^2^ statistics. The I^2^ values were categorized into four groups: of 0%~29%, 30%~49%, 50%~74%, and 75%~100%, representing unimportant, moderate, substantial, and considerable inconsistency, respectively [32,33]. We drew a forest plot to visually represent the effect size of each study and the pooled analyses. Finally, we calculated the publication risk bias using Egger’s test and presented the funnel plot asymmetry visually.

## 3. Results

### 3.1. Study Identification

**Figure 1:**
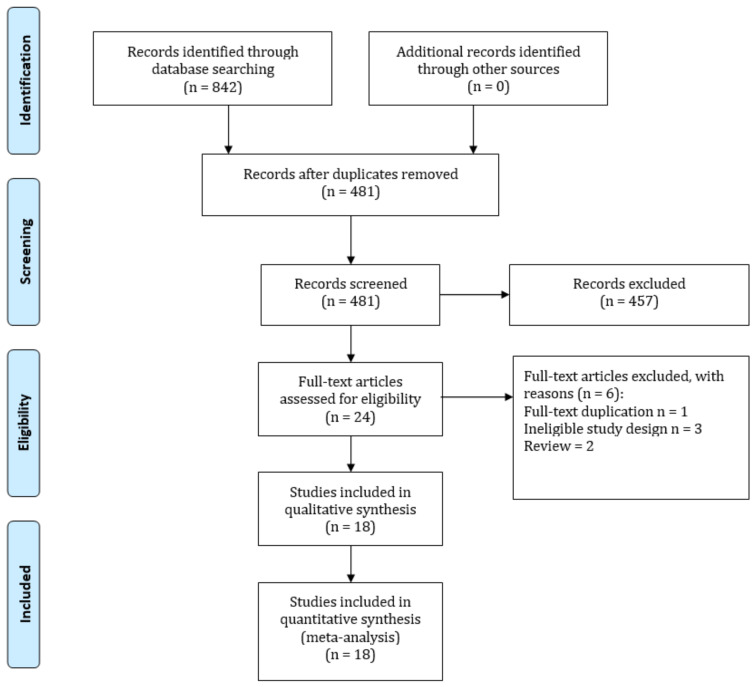
PRISMA diagram of the study selection process.

Figure 1 presents the overall process of study selection using a PRISMA diagram. Our initial search yielded 842 articles, and 361 were removed because they were duplicates. Next, 457 articles were excluded after title and abstract screening. We further screened the reference lists of 24 articles to obtain more potential articles, but no additional articles were identified. A full-text review of 24 articles was conducted. Finally, 18 articles were included in our study [34,35,36,37,38,39,40,41,42,43,44,45,46,47,48,49,50].

### 3.2. Study Characteristics and Quality Assessment

Table 1 shows the characteristics of the included studies. Among the 18 included studies, 10 were cohort studies and 8 were case–control studies. Nine of the studies were from North America, five were from Asia and four were from Europe. The study sample size ranged from 230 to 2,004,692 individuals in total. The studies included 3.7 million individuals with 31,153 PD patients. The average NOS score was 7.33, with an interquartile range (IQR) of 6–9.

### 3.3. Statin Use and PD Risk

**Figure 2:**
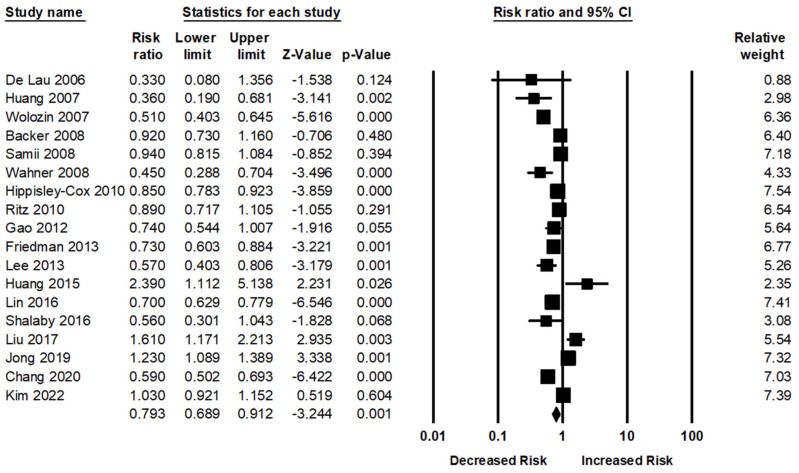
Association between statin use and PD risk [34,35,36,37,38,39,40,41,42,43,44,45,46,47,48,49,50,51,52].

Eighteen studies assessed the association between statin therapy and the risk of PD. Statin use was significantly associated with decreased risk of PD compared with the absence of statin use; the pooled RR was 0.79 (95% CI: 0.68–0.91, *p* = 0.001). Significant heterogeneity was present across the studies (Q = 149.02, *p* < 0.001, I^2^ = 82.05%) in the random-effect models (Figure 2).

### 3.4. Subgroup Analysis

To make the findings more robust, we performed subgroup analyses using various study features (e.g., the influence of study design) and clinical factors (e.g., adjusted for age) (Table 2). Ten cohort and eight case–control studies compared the risk of PD for statin users and non-statin users. The pooled RRs for cohort and case–control studies were 0.75 (95% CI: 0.62–0.91, *p* = 0.004) and 0.85 (95% CI: 0.70–1.04, *p* = 0.09). There was significant heterogeneity among the studies (Q = 98.14, *p* < 0.001, and I^2^ = 90.83% and (Q = 35.35, *p* < 0.001, and I^2^ = 80.19, respectively).

Nine studies from North America evaluated the impact of statin therapy on the risk of PD. The overall pooled RR was 0.81 (95% CI: 0.60–1.09, *p* = 0.17), with significant heterogeneity among the studies (Q = 85.20, *p* < 0.001, I^2^ = 90.61%). However, the pooled RRs for studies from Europe and Asia were 0.85 (95% CI: 0.79–0.92, *p* < 0.001, number of studies, n = 4) and 0.71 (95% CI: 0.57–0.90, *p* = 0.005, n = 5), respectively.

The overall pooled RRs for the risk of PD for high and moderate-quality methodologies were 0.79 (95% CI: 0.64–0.97, *p* = 0.02, n = 10) and 0.79 (95% CI: 0.63–0.98, *p* = 0.03, n = 8), respectively. Fourteen studies were adjusted for age when evaluating the risk of PD among statin users compared to non-users. The pooled RR for studies that adjusted for age was 0.80 (95% CI: 0.66–0.97, *p* = 0.02). Moreover, thirteen studies adjusted for gender and ten studies adjusted for smoking status to assess the risk of PD with statin use. The pooled RRs were 0.84 (95% CI: 0.69–1.03, *p* = 0.06) and 0.79 (95% CI: 0.62–0.97, *p* = 0.02), respectively.

Six studies examined the risk of PD with simvastatin; the pooled RR was 0.67 (95% CI: 0.53–0.84, *p* = 0.001), with a significant heterogeneity (Q = 111.29, *p* < 0.001, I^2^ = 93.71%). The pooled RRs for studies using atorvastatin, lovastatin, and pravastatin were 0.73 (95% CI: 0.59–0.90, *p* = 0.003, n = 5), 0.89 (95% CI: 0.65–1.21, *p* = 0.46, n = 3), and 1.09 (95% CI: 0.65–1.84, *p* = 0.72), respectively.

### 3.5. Sensitivity Analysis

To assess whether gender, the nature of the statins used, or the duration of statin usage substantially influenced the main findings, we calculated the summary effect size and heterogeneity of the main analysis. The pooled RRs for PD risk in male and female users were 0.62 (95% CI: 0.39–0.98, *p* = 0.04, n = 4, Q = 34.44, *p* < 0.001, I^2^ = 88.38%) and 0.47 (95% CI: 0.30–0.73, *p* = 0.001, n = 3, Q = 8.86, *p* < 0.001, I^2^ = 66.16%), respectively. The pooled RRs for PD risk for lipophilic and hydrophilic statin users were 0.86 (95% CI: 0.56–1.34, *p* = 0.52, n = 4, Q = 39.51, *p* < 0.001, I^2^ = 92.40%) and 1.03 (95% CI: 0.86–1.23, *p* = 0.69, n = 4, Q = 2.78, *p* = 0.42, I^2^ = 0%), respectively. Furthermore, we assessed the impact of the duration of statin therapy and the risk of PD. Six studies examined the impact of shorter-duration statin use, defined as the use of statins for <1 year, on the risk of PD, and the pooled RR was 0.94 (95% CI: 0.67–1.31, *p* = 0.73), with a significant heterogeneity (Q = 39.97, *p* < 0.001, I^2^ = 87.47%). In contrast, for longer-duration statin use, defined as the use of statin use for >5 years, the pooled RR was 0.49 (95% CI: 0.26–0.92, *p* = 0.02, n = 2), with no significant heterogeneity (Q = 1.27, *p* = 0.25, I^2^ = 21.59%).

### 3.6. Publication Bias

**Figure 3:**
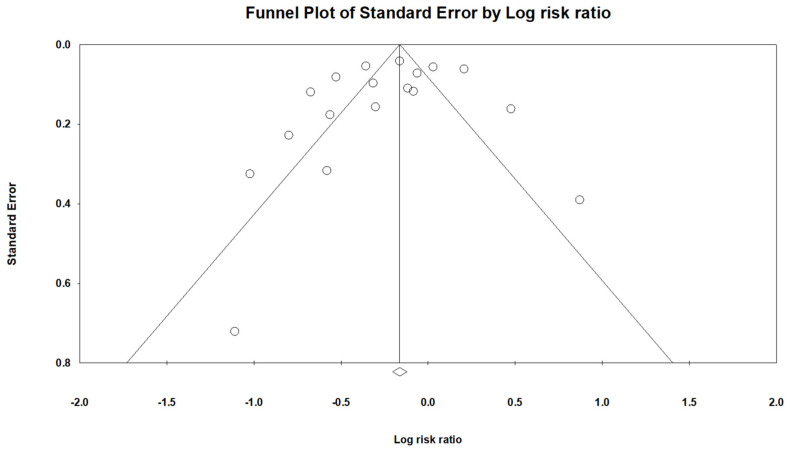
Funnel plot for the detection of possible bias of publication on the effect of statin use on PD risk.

We drew a funnel plot to assess publication bias and did not find any significant bias (Figure 3). In addition, there was no obvious publication bias, assessed by the trim-and-fill method for PD risk.

## 4. Discussion

The present meta-analysis, comprising 18 observational studies with 3.7 million individuals, revealed that statin use was associated with a 19% reduced risk of PD. Our findings should be interpreted with caution because there was significant heterogeneity among the studies and because observational studies are unable to determine whether the association is causal or if there are any other confounding factors. However, for the studies that adjusted for age, gender, and smoking status, the use of statins significantly reduced the risk of PD. Moreover, the use of statins for >5 years significantly decreased the risk of PD, whereas there was no statistically significant decrease in PD among short-term (<1 year) statin users. Furthermore, the use of lipophilic statins was associated with a decreased risk of PD, but there was no association between hydrophilic statin and PD risk. The primary findings of our study are consistent with the previous meta-analyses; that is, overall, the use of statins is associated with a decreased risk of PD.

The previous meta-analysis suggests that statins may be beneficial for reducing the risk of PD [22,23,24,25]. Additionally, both our current study (adjusted RR = 0.79; 95% CI: 0.68–0.91) and previous work by Poly et al. [27] found that statins appear to have strong protective effects against PD (RR = 0.70; 95% CI: 0.58–0.84). However, compared to the study by Poly et al., we added five studies and had more subjects. Moreover, we have provided more comprehensive subgroups and sensitivity analyses to determine whether there is an association between these subgroups and any factors that affect the association. This study included a higher number of studies and estimated the pooled risk ratio using the adjusted effect size from each study; therefore, the association between statin use and PD risk reduction cannot be ruled out.

The exact mechanism describing how statins affect PD risk reduction has not been clearly elucidated, but several possible pathological mechanisms have been proposed. A preclinical study of the PD model showed that statins protected dopamine-containing neurons in the substantia nigra and striatum in the brain of 6-OHDA-lesioned rats. This finding presents an N-methyl-d-aspartate (NMDA) modulatory effect, providing a new paradigm to ameliorate anti-inflammatory activity in PD [51]. Previous studies reported that statins regulate tumor necrosis factor (TNF-a) and matrix metallopeptidase-9 (MMP-9) through NMDA receptor-mediated anti-inflammatory mechanisms [52,53], which ultimately provide neuroprotection against dopaminergic neurodegeneration [54]. Several animal studies have revealed that statin use could attenuate cholesterol metabolites, which help to inhibit α-synuclein aggregation and reduce the toxic effect in dopaminergic neurons [55,56]. Moreover, an in vitro study reported that statins impede neurotoxicity and apoptosis by decreasing reactive oxygen species (ROS) production [57]. Furthermore, a lipopolysaccharide (LPS)-induced rat model of PD demonstrates that statins reduce the risk of PD by promoting neuronal repair and regeneration [58]. Statins also reduce oxidative stress and improve substantia nigra function.

In the subgroup analysis stratified by region, we found a reduced risk of PD with statin therapy in all subgroups, although there was a non-significant reduced risk of PD in patients from North America. This finding can be partially explained by the different races in this region. In support of this finding, previous studies have documented statin response variation among races [59,60]. The specific reasons for this finding are unclear; lifestyle factors such as diet [61,62] and exercise [63] could contribute to the wide confidence interval in the North American group. Age, gender, and smoking status are considered potential confounding factors for PD; however, our study results show that the use of statins was associated with a decreased risk of PD when the analysis was adjusted for these confounding factors. Moreover, the subgroup analysis of high and moderate-quality studies showed a significantly reduced PD risk because these studies adjusted all potential variables during the effect size calculation.

As for individual statin use, simvastatin and atorvastatin showed a reduced risk of PD, whereas pravastatin showed no association with the risk of PD. The classification of statins can be a possible explanation for these variations. Pravastatin is a hydrophilic statin that is distributed mainly in the liver. Lipophilic statins (e.g., simvastatin, atorvastatin) are widely distributed in all tissues [64] and can cross the blood–brain barrier; they potentially affect the central nervous system [65]. Our subgroup analysis shows that simvastatin was more protective than atorvastatin because simvastatin is more lipophilic than atorvastatin [66]. Previous studies have reported that simvastatin is superior at crossing the blood–brain barrier and provides more neuroprotective effects than atorvastatin [66,67].

Our updated meta-analysis has several strengths and limitations. First, this is the most comprehensive meta-analysis performed on this subject; it comprises 18 observational studies with more than 3 million people. Second, our study contains broad subgroup and sensitivity analyses which can be helpful in making a fruitful clinical decision in the real-world clinical setting. Third, our study shows the impact of different types of statins such as atorvastatin and pravastatin on the risk of PD. It also shows the relationship between duration of statin use and PD risk. There were several limitations that need to be addressed. First, there was significant heterogeneity among the studies in the main and subgroup analyses. However, this heterogeneity can be explained by the different study designs, durations, and qualities and by regional effects. Second, our study was unable to show the impact of the statin dose on the risk of PD due to a lack of data. Third, all the included studies were observational studies. As such, there might be several potentially confounding factors, and the findings should be considered with caution.

## 5. Conclusions

The findings of our updated meta-analysis show a significant PD risk reduction due to statin use. Moreover, the long-term use of statins (>5 years) significantly reduced the risk of PD. As the findings are mainly based on limited observational studies, there may be several potential confounding factors and different definitions of statin users and exposure. Therefore, our results should be used with caution; there is not enough statistical power to confirm or refute these observed associations. A study with a large sample size, high-quality methodology, and long follow-up (more than ten years) is needed to either confirm or refute these findings. Moreover, biological studies are required to verify whether a given association is causal or observed. Until higher-quality evidence is available, statins should only be prescribed for their original purpose (reducing cardiovascular risk); they should not be repurposed for PD risk reduction.

## Figures and Tables

**Table 1:** Basic characteristics of included studies [34,35,36,37,38,39,40,41,42,43,44,45,46,47,48,49,50,51,52].

Study	Publication Year	Study Duration	Study Type	Population	PD Cases	Definition of Statin Use	Identification of Statin Users	Country	Quality Score
De Lau	2006	1990–2004	Co	6465	87	Medical record	ATC	Netherland	8
Huang	2007	2002–2004	C-C	236	124	Medical record	ATC	USA	7
Wolozin	2007	2003–2005	Co	1,226,198	5107	Database	ATC	USA	6
Becker	2008	1994–2005	C-C	7274	3637	Medical records	ATC	UK	9
Samii	2008	1997–2003	C-C	23,780	4756	Medical records	ATC	Canada	6
Wahner	2008	2001–2007	C-C	654	312	Self-report	ATC	USA	7
HippisleyCox	2010	2002–2008	Co	2,004,692	3553	Database	ATC	UK	6
Ritz	2010	2001–2006	C-C	11,582	1931	Medical report	ATC	Denmark	7
Gao	2012	1994–2006	Co	129,006	644	Self-report	ATC	USA	7
Friedman	2013	2000–2007	Co	87,971	824	Database	ATC	Israel	8
Lee	2013	2001–2008	Co	43,810	1886	Database	ATC	China	9
Huang	2015	1987–2008	Co	15,291	56	Medical records	ATC	USA	6
Lin	2016	1996–2008	Co	50,432	651	Database	ATC	China	8
Shalaby	2016	2009–2014	C-C	230	108	Self-report	ATC	USA	7
Liu	2017	2008–2012	C-C	4644	2322	Database	ATC	USA	7
Jong	2019	2002–2015	Co	76,043	1427	Database	ATC	USA	8
Chang	2020	1996–2013	Co	48828	692	Database	ATC	Taiwan	8
Kim	2022	2002–2015	C-C	15,130	3036	Database	ATC	South Korea	8

Note: C-C = case–control study; Co = cohort study; ATC = Anatomical Therapeutic Chemical Classification System.

**Table 2:** Subgroup analysis.

Study	No of Studies	Pooled Estimates		Test of Heterogeneity		
		RR (95% CI)	*p*-Value	Q Value	*p* Value	I^2^ (%)
All studies	18	0.79 (0.68–0.91)	0.001	149.02	<0.001	82.05
Study design						
Cohort	10	0.75 (0.62–0.91)	0.004	98.14	<0.001	90.83
Case–control	8	0.85 (0.70–1.04)	0.09	35.35	<0.001	80.19
Region						
North America	9	0.81 (0.60–1.09)	0.17	85.20	<0.001	90.61
Europe	4	0.85 (0.79–0.92)	<0.001	2.26	<0.001	0
Asia	5	0.71 (0.57–0.90)	0.005	42.86	<0.001	90.66
Adjusted for age						
Yes	14	0.80 (0.66–0.97)	0.02	125.79	<0.001	89.66
No	4	0.75 (0.61–0.92)	0.006	21.36	<0.001	85.96
Adjusted for gender						
Yes	13	0.84 (0.69–1.03)	0.06	102.57	<0.001	88.30
No	5	0.69 (0.58–0.82)	<0.001	21.91	<0.001	81.74
Adjusted for smoking status						
Yes	10	0.78 (0.62–0.97)	0.02	86.14	<0.001	89.14
No	8	0.79 (0.65–0.96)	0.02	56.60	<0.001	87.63
Quality of Studies						
Medium	10	0.79 (0.64–0.97)	0.02	59.85	<0.001	84.96
High	8	0.79 (0.63–0.98)	0.03	88.93	<0.001	92.12
Statin type						
Simvastatin	6	0.67 (0.53–0.84)	0.001	111.29	<0.001	93.71
Atorvastatin	5	0.73 (0.59–0.90)	0.003	32.80	<0.001	81.71
Lovastatin	3	0.89 (0.65–1.21)	0.46	6.26	0.005	81.02
Pravastatin	3	1.09 (0.65–1.84)	0.72	6.56	0.08	54.30
Study year						
≤5	3	0.68 (0.28–1.65)	0.39	37.41	<0.001	94.65
≤10	7	0.76 (0.66–0.88)	<0.001	18.61	0.005	67.77
>10	8	0.88 (0.69–1.11)	0.29	87.86	<0.001	92.03

## Data Availability

Not applicable.
